# Pazopanib-Induced Alopecia, an Underestimated Toxicity?

**DOI:** 10.3389/fonc.2015.00112

**Published:** 2015-05-11

**Authors:** Andrea Biondo, Helen Alexander, Komel Khabra, Lisa Pickering, Martin Gore, James Larkin

**Affiliations:** ^1^Royal Marsden Hospital, London, UK

**Keywords:** pazopanib, sunitinib, metastatic renal cancer, alopecia, toxicity

## Abstract

Pazopanib and sunitinib are treatment options for metastatic renal cell cancer (mRCC), with similar efficacy, and minor differences in their toxicity profile. Our experience has suggested that pazopanib-induced alopecia may be a potentially significant but previously under-reported toxicity. For this reason, we performed a retrospective review of the clinical records of all patients with mRCC treated with pazopanib at the Royal Marsden Hospital from European licensing until June 2013, and all patients treated with sunitinib over the same period. We found that 36 patients with mRCC were treated with pazopanib and 85 patients with sunitinib. Four of the 36 (11%) patients treated with pazopanib developed alopecia severe enough to warrant a wig versus none of 85 patients treated with sunitinib (*p* = 0.007). In conclusion, grade 2 pazopanib-induced alopecia was reported at significantly higher rates when compared to sunitinib-induced alopecia. Hence, in our view, patients should be informed about this potential toxicity when discussing the treatment options for mRCC.

## Introduction

Pazopanib and sunitinib are both approved first line treatments for metastatic renal cell cancer (mRCC) (1). They appear to have similar efficacy, with minor differences in their toxicity profile (2, 3). In the PISCES study, a randomized double-blind cross-over trial, patients showed a preference for pazopanib because of less fatigue and better overall quality of life. The COMPARZ study, a randomized open-label trial of pazopanib versus sunitinib in the first line treatment of mRCC, showed non-inferiority of progression-free survival (PFS) with pazopanib and favored its quality of life and safety profiles. In current clinical practice, the choice of treatment is at the discretion of the treating physician and patient.

Anti-cancer treatment-induced alopecia may impact significantly on the quality of life of patients (4). Anecdotal evidence in our practice has suggested that pazopanib-induced alopecia may be a potentially significant but previously under-reported toxicity. We therefore retrospectively analyzed rates of pazopanib-induced alopecia, severe enough to warrant a wig [i.e., Common Terminology Criteria for Adverse Events (CTCAE) grade 2; Table 1], in comparison to the rates of sunitinib-induced alopecia in patients treated for mRCC.

**Table 1:** **Alopecia grading, adapted from CTCAE criteria (5)**.

Adverse event	Grade				
	1	2	3	4	5
**CTCAE v3.0**					
Alopecia	Thinning or patchy	Complete	–	–	–
**CTCAE v4.0**					
Alopecia	Hair loss of <50% of normal for that individual that is not obvious from a distance but only on close inspection; a different hair style may be required to cover the hair loss but it does not require a wig or hair piece to camouflage	Hair loss of ≥50% normal for that individual that is readily apparent to others; a wig or hair piece is necessary if the patient desires to completely camouflage the hair loss; associated with psychosocial impact	–	–	–

## Materials and Methods

We performed a retrospective review of the clinical records of all patients with mRCC treated with pazopanib at the Royal Marsden Hospital from European licensing until June 2013, and all patients treated with sunitinib over the same period. Fisher’s exact statistical test was used to compare the two groups, and the Kaplan–Meier method and log rank test for PFS analysis. PFS was defined as the date of starting treatment to the date of progression.

## Results

**Figure 1:**
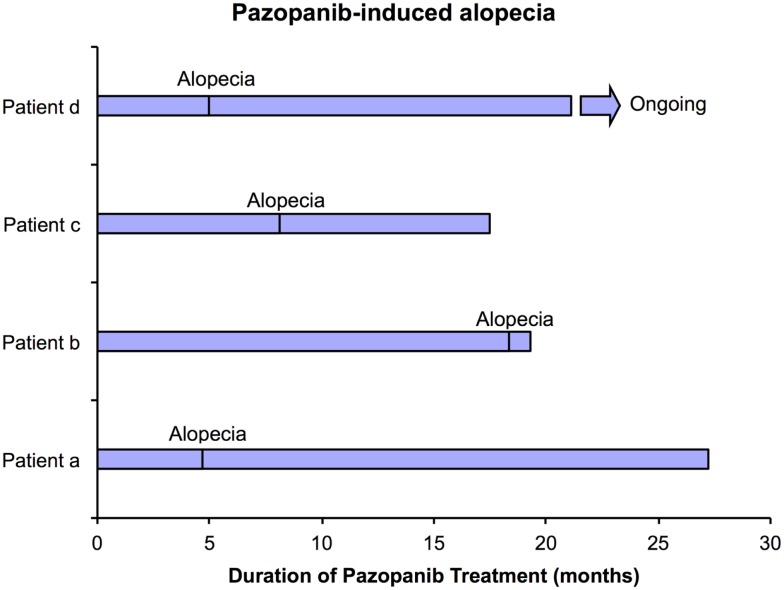
**Timeline for the development of alopecia in 4 patients treated with pazopanib**.

Thirty-six (F = 13, M = 23; median age 64.8 years) patients with mRCC were treated with pazopanib and followed up from November 2010 to June 2013, and eighty-five (F = 26, M = 59; median age 66.2 years) patients with mRCC were treated with sunitinib over the same period. Four of the 36 (11%) patients treated with pazopanib followed up at the Royal Marsden developed alopecia severe enough to warrant a wig (Figure 1) versus none of 85 patients treated with sunitinib (*p* = 0.007). All four patients who developed alopecia while on pazopanib were females (31% of females on pazopanib), aged 88, 75, 70, and 67 years at the start of treatment. None of the four patients had any reported degree of alopecia at baseline and none had significant thyroid dysfunction while on therapy. The median time for the development of alopecia while on pazopanib was 6.5 months (range 4.7–18.3) from the start of therapy. Patients a, b, and c did not receive any radiotherapy to the brain, but patient d started treatment with pazopanib in October 2012 and had treatment with Cyberknife radiosurgery at the same time (20 Gy in one fraction to three right temporal lobe lesions) and in February 2014 (24 Gy in one fraction to a right frontal lobe lesion). Alopecia was first noted in March 2013. If we exclude patient d from the analysis, the difference in pazopanib-induced alopecia versus sunitinib remains statistically significant.

The median PFS on pazopanib was 6.6 months [95% confidence interval (CI) 2.1–10.1] versus 5.3 months on sunitinib (95% CI 3.4–7.8; *p* = 0.583).

## Discussion

In this retrospective analysis, grade 2 pazopanib-induced alopecia was reported at significantly higher rates when compared to sunitinib-induced alopecia.

The CTCAE are generally used in clinical trials to grade the severity of adverse events, including drug-induced alopecia (Table 1). In the COMPARZ study (3), comparing pazopanib to sunitinib in the first line treatment of mRCC, patients were followed up until death or withdrawal from the study. Patients in the pazopanib group showed a statistically significant higher risk of developing any grade of alopecia versus the sunitinib group (14 versus 8%). The grade 1 and 2 rates of alopecia were, however, grouped together. In the PISCES study (2), patients were assessed for adverse events over a 10-week period on each drug. Drug-induced alopecia was not reported in this trial for either drug, probably due to the short follow-up period on each drug.

In this retrospective study, we make the assumption that the development of significant alopecia on treatment was accurately recorded and that all potential confounding factors were considered. Cyberknife to the brain may cause a degree of alopecia. No concomitant drugs recorded were deemed to be a likely cause for the grade 2 alopecia.

In our analysis, the median time for development of alopecia was 6.5 months. The median PFS with pazopanib in our retrospective analysis was 6.6 months. The median PFS reported in the much larger COMPARZ study is 8.4 months (3). This makes pazopanib-induced alopecia a potentially clinically significant chronic toxicity, which may have been thus far underestimated. In our view, patients should be informed about this potential toxicity when discussing the treatment options for mRCC.

## Conflict of Interest Statement

Lisa Pickering: Consulting or advisory role: Novartis, Pfizer, Astellas Pharma, GlaxoSmithKline, Janssen-Cilag, Sanofi-Aventis, Dendreon; Travel, accommodations, expenses: Astellas Pharma, Janssen-Cilag. James Larkin: Research funding: Pfizer, Novartis. Andrea Biondo, Helen Alexander, Komel Khabra and Martin Gore have no conflicts of interest to declare.
